# Predicting Substance Use in Young Adults: The Role of Childhood Adversity

**DOI:** 10.3390/medicina62040772

**Published:** 2026-04-16

**Authors:** Liudas Vincentas Sinkevicius, Sandra Sakalauskaite, Mykolas Simas Poskus, Danielius Serapinas

**Affiliations:** 1 Institute of Psychology, Mykolas Romeris University, 08303 Vilnius, Lithuaniad_serapinas@mruni.eu (D.S.); 2 Department of Health Psychology, Lithuanian University of Health Sciences, 50161 Kaunas, Lithuania; 3 Laboratory of Immunology, Department of Immunology and Allergology, Lithuanian University of Health Sciences, 50161 Kaunas, Lithuania; 4 Department of Family Medicine, Lithuanian University of Health Sciences, 50161 Kaunas, Lithuania

**Keywords:** adverse childhood experiences, alcohol, HPA, inflammation, kynurenine pathway, marijuana, substance abuse, substance use

## Abstract

*Background and Objectives*: One of the strongest early factors influencing later psychoactive substance use is adverse childhood experiences (ACEs). Studies investigate a variety of adverse experiences in relation to substance use, yet not all adverse childhood experiences are equal in intensity and harm. Our study aimed to address this gap by examining in detail the associations between individual ACEs, broader ACE categories, and different forms of psychoactive substance use. *Materials and Methods*: The study included 709 participants who completed self-report questionnaires. ACEs were measured using the MACE questionnaire. Marijuana use was measured using the CUDIT-R, alcohol use using the AUDIT, and heavy psychoactive substance use using the ASSIST. Linear regression analyses were used to predict associations. As expected, only a small part of the sample reported hard drug use; some analyses are limited to substantially fewer observations. *Results*: All regression models were statistically significant and predicted all three categories of psychoactive substances, but if we count the individual adverse experiences, the results become different. Although the results showed that ACE is a significant predictor of hard drug use and explains 25% of the variance, it is separately only emotional neglect that is associated with hard drug use. The regression analysis also explains 14% of the variance in marijuana use, but when considered separately, we found associations only with emotional neglect. The severity of alcohol use explains 13% of the variance, but only a few ACEs reach statistical significance: peer physical bullying, physical violence, and sexual abuse. *Conclusions*: The findings of our study suggest that adverse childhood experiences may not be qualitatively equivalent and therefore may not be evaluated only as a cumulative risk score. Separate ACE evaluations, instead of aggregate calculation of ACEs, may be useful to understand better which specific negative experiences have the greatest impact on subsequent use of psychoactive substances. The regression models explain only a small portion of the variance, which suggests that other factors may contribute to a larger share.

## 1. Introduction

Around the world, dominant fatal diseases have changed from infectious to autoimmune, heart diseases, etc., over the past few hundred years [1]. As the situation has changed, mental disorders such as depression and anxiety are also influencing the mortality and disability of the population. With the rapid increase in depression and anxiety disorders in recent decades, people instinctively look for ways to improve their mental states, and this short-term relief often comes from psychoactive substances [2]. People have used psychoactive substances for thousands of years, but only in the last century, under the influence of industrialization, the use of psychoactive substances has gained significant momentum and in some countries, epidemics have been declared [3]. Although one of the biggest reasons cited is accessibility, of all those who use psychoactive substances, only about 10–20% become addicted, so the question naturally arises as to what is special about this group of people [4,5].

In primary psychological theories, many authors viewed humans in a rather deterministic way: their behavior was attributed to early experiences, subconscious conflicts, and internal mental mechanisms [6]. Adverse childhood experiences can also be interpreted in psychological theories that emphasize different mechanisms through which early experiences can influence later substance use. For example, the social learning theory associated with Albert Bandura states that behavior is shaped by observation, imitation, and reinforcement. This perspective explains how the use of psychoactive substances by parents or other closely associated people from one’s childhood can become a pattern that later manifests itself in adult behavior [7]. Meanwhile, attachment theory, developed by John Bowlby, would explain this phenomenon by focusing on the quality of early relationships with caregivers [8]. Insecure attachment styles are associated with specific internal working models about one’s self and others, which may be related to difficulties in emotion regulation, impulsivity, and a lack of adaptive self-regulation strategies [9,10,11]. In this case, the use of psychoactive substances would be understood as a way of regulating emotions or reducing psychological distress. This is also supported by studies showing the association between insecure attachment styles and psychoactive substance use, as well as studies explaining this through self-regulation and self-healing mechanisms [11,12].

The human desire to use psychoactive substances is explained by various disciplines, one of which is genetics, which simply destroys John Locke’s famous phrase that man is a Tabula Rasa. After extensive animal and human studies, it is suggested that genetic and epigenetic changes in the dopaminergic system can result in dysfunction and an inadequacy in the neurotransmission of reward or pleasure (described as Reward Deficiency Syndrome, RDS). These changes are associated with a higher risk of addiction. The reduced ability to experience satisfaction from daily activities stimulates the search for new stimuli. Therefore, young individuals may be more prone to risky and early use of psychoactive substances. In addition, RDS may be associated with various psychological consequences, such as attention-deficit hyperactivity disorder (ADHD), depression, and anxiety disorders, as well as difficulties in regulating emotions and lower self-esteem [13,14].

Recently, the study of addictions has focused increasingly on neuroimmune mechanisms that link stress and addiction [15]. The HPA axis (hypothalamic–pituitary–adrenal) and the immune system form a neuroendocrine-immune network that responds to stress and psychoactive substances, highlighting a complex interaction that is crucial for understanding the biological basis of addiction [16,17]. Addictive substances activate the HPA axis (secrete cortisol) and alter the immune response. Chronic stress disrupts both systems, reducing stress resilience, altering brain functions (e.g., decision-making, substance use, and relapse), and increasing disease susceptibility [18]. Chronic stress and psychoactive substances activate innate immune cells in the brain, promoting neuroinflammation and cytokine release [19]. These inflammatory processes can disrupt prefrontal cortex function, impair self-control, and promote psychoactive substance use [18]. At the same time, alcohol and opioids further destabilize the immune system, increasing somatic vulnerability and the risk of infections, thus emphasizing the nature of addiction as a systemic disease [20].

**Figure 1:**
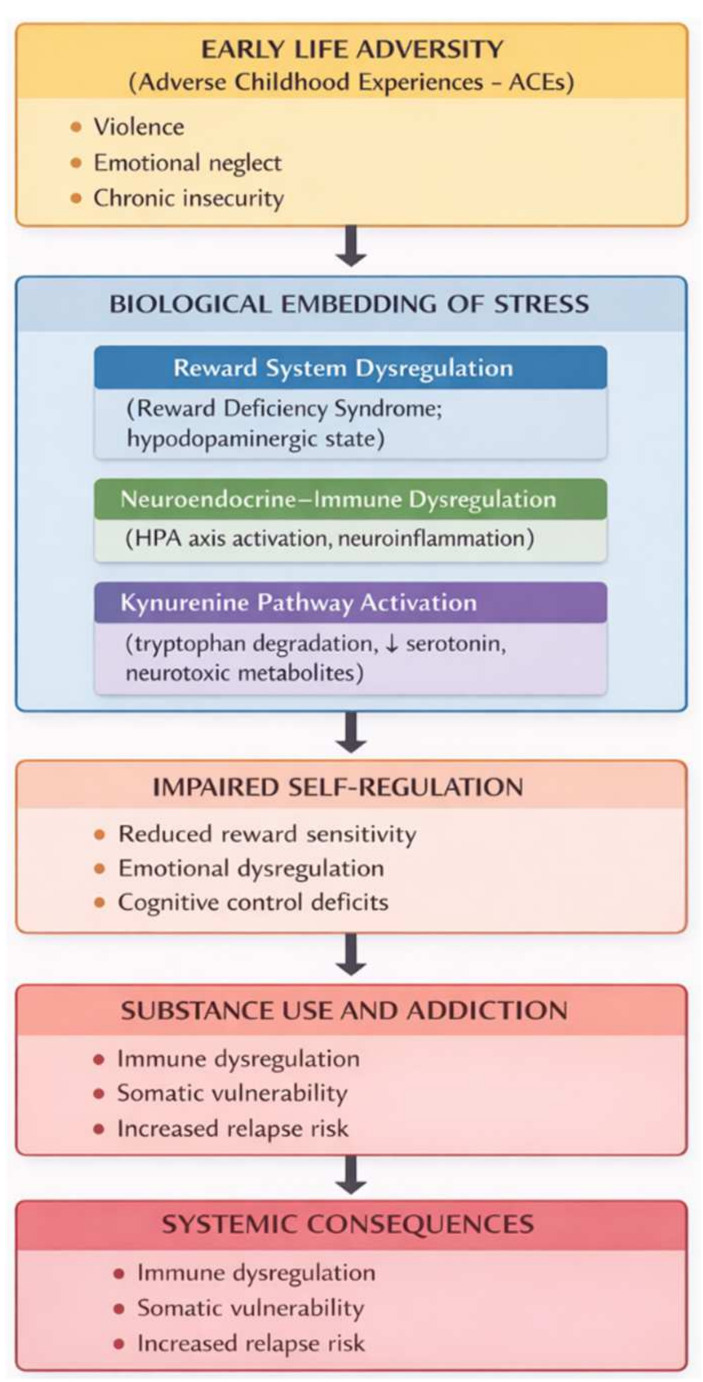
Pathways linking adverse childhood experiences to addiction vulnerability.

In the context of addiction, the most common focus is on the important role of cortisol, a stress hormone that affects not only neural plasticity but also the immune response. However, in recent years, there has been a significant increase in interest in the serotonergic system, which contributes to the formation of drug use-associated behavior and the transition to addiction, including many of the following substances: cocaine, amphetamine, methamphetamine, morphine/heroin, cannabis, alcohol and nicotine [21,22,23]. The kynurenine pathway (KP) mechanism is important here, being associated with depression and addictive behavior [24,25]. Research suggests that CP metabolism plays a role in the metabolic circuit connecting the immune system (inflammatory processes, the HPA axis, and neurotransmitter balance), which is the basis of addictive behavioral patterns, especially in alcohol use disorders. The problem is that the use of psychoactive substances activates tryptophan-degrading enzymes (indolamine 2,3-dioxygenase (IDO) and tryptophan 2,3-dioxygenase (TDO), which accelerate the conversion of tryptophan to KP metabolites [26]. This process reduces the amount of tryptophan needed for serotonin synthesis and promotes the production of toxic metabolites. Such a cycle affects the immune system by suppressing T lymphocyte activity, promoting immune tolerance, and reducing serotonin synthesis [24,27]. This explains and enhances the risk of emotional and cognitive disorders, thus contributing to the detection and relapse of SUD symptoms [25]. Based on this knowledge, it can be assumed that early stress is not limited to psychological consequences alone—it leaves a long-term biological footprint, altering the trajectories of stress regulation, the immune system, and brain development (Figure 1). Psychologically, early adversity is associated with emotional and behavioral difficulties, including symptoms of depression, anxiety disorders, and post-traumatic stress disorder, as well as impaired emotion regulation, heightened stress reactivity, difficulties in interpersonal relationships, and lower self-esteem [28,29]. These outcomes are commonly explained by theoretical frameworks such as attachment theory, the stress–diathesis model, and neurodevelopmental models [30].

Thus, when analyzing the social and physiological causes of addiction, it can be stated that adverse childhood experiences (ACEs) play a very important role, which are “recorded” at the biological level. For this reason, it is increasingly being said around the world that negative childhood experiences, such as violence, emotional neglect, or chronic insecurity, affect not only psychological but also physiological health and are recognized as early risk factors that form an increased vulnerability to the development of addiction in later life [31,32,33,34]. Nevertheless, the associations between different ACEs and substance use remain controversial [35,36,37,38,39]. Different ACE types do not all show the same strength or even direction of association with every substance (alcohol, tobacco, cannabis, “hard” drugs, prescription misuse, etc.). Some studies, for example, find strong links between certain abuses and illicit drug use, but some find no links or weak links. So, this does not mean there is no link between adversity and substance use at all, but rather that there is still scientific debate and inconsistent evidence about these links. Still, ACEs are a very broad construct, the factors of which are often summed up, so such a wide variety of negative factors must have one or another consequence. It is very important to understand that a person naturally has to look for ways to regulate the unpleasant states caused by ACEs with legal or illegal substances. Therefore, this study aims to analyze what part of the prevalence of psychoactive substance use can be explained by negative childhood experiences.

## 2. Materials and Methods

### 2.1. Participants and Procedure

The study was conducted in Lithuania, a Central European country. Data were collected using the Google Forms platform in September–November 2024, when the study participants voluntarily completed the questionnaire. All participants were first informed of the study’s purpose, indicated their agreement to participate, and were informed that they could leave the study at any time without completing it. Requests to fill out the questionnaire and share it with friends were sent to prospective study participants. The e-mail databases of two Lithuanian universities, Mykolas Romeris and the Lithuanian University of Health Sciences, were used to distribute the questionnaire. Only individuals aged 18–29 could participate in the study, but more than half were very young, i.e., between 18 and 22 years old. The total sample consisted of 709 study participants, most of whom were women (521) and a smaller proportion were men (188). At the end of the questionnaire, study participants were provided with contact details for psychological helplines, if necessary.

### 2.2. Measurements

#### 2.2.1. Demographic Variables

Minimal demographic information was collected, i.e., biological sex and age. Genealogical questions were also added to the demographic questions: “Did your grandfather/grandmother smoke cigarettes?”; “Did your father/mother smoke cigarettes?”; “Do you think/know that your grandfather/grandmother was addicted to alcohol/other psychoactive substances?”; “Do you think/know that your father/mother was addicted to alcohol/other psychoactive substances?”. The scale demonstrated reasonable internal consistency: KR-20 = 0.573.

General questions about smoking without distinguishing products were also added: “Do you smoke?”; “If so, how old were you when you started smoking?”. This question is coded as D5 in the results.

#### 2.2.2. Adverse Childhood Experiences (ACEs)

The standard ACE questionnaire [40] used in the pilot study was not suitable for our sample due to cultural differences when we received feedback from respondents. Our pilot study participants reported that it would be useful to add peer influence, which is not included in the standard ACEs questionnaire, and some questions were completely irrelevant, such as whether a family member has been imprisoned, which is a very rare occurrence in Lithuania. The study participants also criticized the ACE questionnaire for ambiguous questions that were difficult to answer unambiguously. Physical and emotional neglect (Neglect), sexual, physical and emotional violence (Abuse), witnessing interpersonal violence, witnessing violence to siblings, peer emotional abuse, and peer physical bullying were assessed using the MACE (Maltreatment and Abuse Chronology of Exposure (MACE) questionnaire [41]). The MACE questionnaire consists of 58 interval statements, which ask about events that occurred before adulthood and are assessed on a 5-point Likert scale. Subscales used: (WIV) witnessing interpersonal violence (α = 0.867; Ω = 0.876) (e.g., “Saw adults living in the household hit your mother (stepmother, grandmother) so hard, or intentionally harm her in some way, that she received or should have received medical attention.”); (SA) sexual abuse (α = 0.801; Ω = 0.830) (e.g., “Actually had sexual intercourse (oral, anal or vaginal) with you.”); (PPM) parental physical maltreatment (physical abuse) (α = 0.883; Ω = 0.890) (e.g., “Hit you so hard that it left marks for more than a few minutes.”); (PPN) physical neglect (α = 0.736; Ω = 0.753) (e.g., “You had to wear dirty clothes”); (PEN) parental emotional neglect (α = 0.734; Ω = 0.769) (e.g., “A parent or other important parental figure did not have the time or interest to talk to you.”); (PVA) parental verbal abuse (α = 0.884; Ω = 0.886) (e.g., “Said hurtful things that made you feel bad, embarrassed or humiliated more than a few times a year.”); (NVEA) non-verbal emotional abuse (α = 0.778; Ω = 0.777) (e.g., “Parent no time or interest”); (WVS) witnessing violence to siblings (α = 0.666; Ω = 0.771) (e.g., “Parents or adults living in house hit your sibling (stepsibling) so hard that it left marks”); (PEA) peer emotional abuse (α = 0.911; Ω = 0.913) (e.g., “Swore, called you names/insults more than few times per year”); (PPB) peer physical bullying (α = 0.832; Ω = 0.844) (e.g., “Threatened you in order to take money or possessions”). Participants rated all items on a five-point Likert scale from 0 (never) to 4 (very often). All scales show good internal consistency.

#### 2.2.3. The Alcohol Use Disorders Identification Test (AUDIT)

The Alcohol Use Disorders Identification Test (AUDIT) was used to measure the severity of alcohol use. The test was developed by the World Health Organization (WHO) [42] to measure the risk of alcohol use. The questionnaire consists of 10 questions that ask about consumption in the past 12 months: “How often do you have six or more drinks on one occasion?”; “How often do you have a drink containing alcohol?”. The questions are measured on a 5-point Likert scale, and the total score can be used to measure the severity of alcohol use (α = 0.855; Ω = 0.866).

#### 2.2.4. The Cannabis Use Disorders Identification Test—Revised (CUDIT-R)

The Cannabis Use Disorders Identification Test—Revised (CUDIT-R) [43] was used to assess cannabis use severity. This test consists of ten questions that measure past cannabis use in the past six months (e.g., “How often do you use cannabis?”; “How many hours were you “stoned” on a typical day when you had been using cannabis?”). The questions are rated on a 5-point Likert scale, the total score of which can be used to measure cannabis use severity (α = 0.909; Ω = 0.919).

#### 2.2.5. The Alcohol, Smoking and Substance Involvement Screening Test (ASSIST)

The Alcohol, Smoking and Substance Involvement Screening Test (ASSIST) was used to assess the use of heavy psychoactive substances [44]. The questionnaire is modified according to the need to investigate the use of different substances. Therefore, in this study, only heavy substances were selected, i.e., hallucinogens, amphetamine, sedatives, cocaine, opioids, inhalants, and new psychoactive substances. The questionnaire assesses the use of psychoactive substances over the past three months and is designed to assess both the risk of use (low, moderate and high) and to create an overall score by which we can monitor the severity of use (α = 0.869; Ω = 0.876).

### 2.3. Analysis Strategy

We used Jamovi 2.6.44 (The jamovi project, 2024) for all analyses, retrieved from [45]. While we did not expect normality in the data because of the nature of the variables being measured, we opted to run regression analyses to investigate the relationship between adverse experiences and substance use, because regression analysis tends to be quite robust against violations of normality [46]. While structural equation modeling could be a more appropriate choice, the low rate of hard drug use among the participants, resulting in a lot of missing values, prevented us from using structural equation modeling, which is generally considered a method for large samples. Participants that did not report use of the substances being investigated were not included in the models. All regression models were investigated for collinearity and none showed signs of VIF < 4. Q-Q plots of standardized residuals were investigated visually and showed signs of non-normality, especially at the tail ends of the distributions. However, even when taking the aforementioned limitations to mind, the regression models remain informative.

## 3. Results

### 3.1. Links Between Childhood Adversity and Substance Use

#### 3.1.1. Descriptive Statistics of the Model Variables

Descriptive statistics for all variables used in the model are presented in Table 1. All variables in alcohol abuse were found to be interrelated with low-to-middle effects. Variables in cannabis abuse were found to be significant in sexual abuse, witnessing violence to siblings, and peer physical bullying. Variables in hard drug abusers were all found to be significant except emotional abuse. As expected, all variables have acceptable skewness and kurtosis to approximate normality, except sexual abuse, which does not follow a normal distribution, and witnessing violence against siblings because of the relative rarity of cases from the results.

It must be noted that very few participants reported hard drug or cannabis use, and therefore, the models for these substances are based on a substantially smaller number of observations than the overall sample of the study (number of missing cases visible in Table 1). Models were computed for participants who reported at least some use of the substances being investigated, while non-users were excluded from the analyses. Therefore, we caution the reader to consider the sample sizes of the presented models.

#### 3.1.2. A Regression Model for Predicting Hard Drug Use

The results presented in Table 2 show that the regression model is appropriate and statistically significant (F(10,146) = 4.92, *p* < 0.001). The coefficient of determination R^2^ = 0.252 statistically explains 25.2 percent of the variation in hard drug abuse, which indicates the average explanatory power of the model. The results obtained reveal that emotional neglect statistically significantly predicts hard drug abuse (*p* < 0.001), i.e., as emotional neglect increases in childhood, hard drug abuse also increases (beta = 0.46921). All other negative childhood experiences were not statistically significant and do not predict hard drug abuse.

#### 3.1.3. A Regression Model for Predicting Cannabis Use

The results presented in Table 3 show that the regression model is appropriate and statistically significant (F(10,134) = 2.23, *p* < 0.019). The coefficient of determination R^2^ = 0.143 statistically explains 14.3 percent of the variation in cannabis abuse, which indicates the average explanatory power of the model. The results obtained reveal that non-verbal emotional abuse statistically significantly negatively predicts cannabis abuse (0.029), i.e., as non-verbal emotional abuse increases during childhood, cannabis abuse decreases (beta = −0.31143). Emotional neglect also statistically significantly predicts cannabis abuse (0.003), i.e., as emotional neglect increases, cannabis abuse also increases (0.45825). All other negative childhood experiences were not statistically significant and do not predict cannabis abuse.

#### 3.1.4. A Regression Model for Predicting Alcohol Abuse

The results presented in Table 4 show that the regression model is appropriate and statistically significant (F(10,599) = 9.04, *p* < 0.001). The coefficient of determination R^2^ = 0.131 statistically explains 13.1 percent of the variation in alcohol abuse, which indicates the average explanatory power of the model. The results obtained reveal that emotional abuse statistically significantly predicts alcohol abuse negatively (0.035), i.e., as emotional abuse increases in childhood, alcohol abuse decreases (beta = −0.13527). On the contrary, sexual abuse statistically significantly predicts alcohol abuse (0.024), i.e., as sexual abuse increases, alcohol abuse also increases (0.11258). Peer physical bullying statistically significantly predicts alcohol abuse (0.041), i.e., as peer physical bullying increases, alcohol abuse also increases (0.12342). Physical maltreatment statistically significantly predicts alcohol abuse (<0.001), i.e., as physical maltreatment increases, so does alcohol abuse (0.21066). All other negative childhood experiences were not statistically significant and do not predict alcohol abuse.

## 4. Discussion

The contribution of individual ACEs to influence on the use of particular psychoactive substances remains limited. Our study was designed to address this gap by examining in detail the associations between individual ACEs, broader ACE categories, and different forms of psychoactive substance use. To our knowledge, this is one of the first studies in Lithuania to investigate these relationships in a large sample of young adults (N = 709). Using validated instruments, including the AUDIT, CUDIT-R, ASSIST questionnaires, together with linear regression analyses, we determined how distinct forms of childhood adversity are associated with alcohol, cannabis, nicotine, and other psychoactive substance use outcomes. We found that all regression models were statistically significant and predicted all three categories of psychoactive substances, but if we count the individual adverse experiences, the results become different. Although the results showed that ACEs are a significant predictor of hard drug use and explains 25% of the variance, it is specifically only emotional neglect that is associated with hard drug use. The regression analysis also explains 14% of the variance in marijuana use, but when considered separately, we found associations only with emotional neglect. The severity of alcohol use explains 13% of the variance, but only a few ACEs reach statistical significance—peer physical bullying, physical violence, and sexual abuse.

Although the use of psychoactive substances is often associated with the desire to regulate emotional states, according to systematic reviews, the use of psychoactive substances among young people is encouraged not only by internal psychological motives, but also by social and environmental factors: social pressure, the desire to fit, curiosity and searching for new experiences. Academic stress, loneliness, and difficulties in adapting to a new social environment also play a significant role. In addition, hedonic motives (the pursuit of pleasure) can determine the use, as well as attempts to overcome, psychological difficulties or negative emotional states. Therefore, psychoactive substances should be understood as a multifaceted phenomenon shaped by interacting biological, psychological, and social factors [47]. The pharmacological properties of different psychoactive substances and their availability are risk factors for addiction, and they also determine how long it takes for use to develop into addiction [48,49]. In this context, psychoactive substances may function as a form of self-medication [50], and individuals exhibiting such patterns appear to be at increased risk of developing addiction [51]. Considering that the majority of mental disorders, including addiction syndrome, appear before the age of 25 [52], and young people reach the peak of the riskiest behavior at the age of 15–19 [53], this highlights the importance of assessing the prevalence of risky use among young adults and identifying the potential early ACE prognostic factors for the development of psychoactive substance use in later life. Our study results of the regression analysis showed that adverse childhood experiences may be considered for hard drug use and explain 25% of the variance in this variable. Additionally, in line with other authors, only emotional neglect, commonly defined as the absence of adequate emotional support and care, has been statistically significantly linked with hard drug use [54]. According to previous studies, it is suggested that certain substances, mostly heroin and hallucinogens, may be related to subjective experiences of emotional relief, increased connectedness, or altered affective states [55]. This phenomenon thought to be associated with neurobiological mechanisms including the effect of heroin on opioid receptors and the endogenous opioid system, reducing not only physical but also emotional pain and causing a feeling of warmth, love and security [56,57]. Although marijuana is illegal in Lithuania and is not distinguished as a “soft” drug, it was included as a separate category in this study due to its high prevalence among young adults. The results of the regression analysis show that adverse childhood experiences explain 14% of the variation in marijuana use. However, when evaluating experiences separately, only emotional neglect may be related to increased marijuana use in adulthood, although many authors find connections with other ACEs [54,58,59,60,61,62].

Adverse childhood experiences can be divided into two large blocks, i.e., direct impact (neglect or abuse) and indirect (family dysfunction). They are shaped by a range of interacting individual and environmental factors. Direct forms of adversity are often associated with parental factors, including mental health difficulties (depression, substance use disorder, a history of trauma, impaired emotion regulation, and high levels of stress or social isolation). The main reasons for indirect factors are usually social conditions, such as lower socioeconomic status, parental absence, family conflict, or parental unavailability [63]. Most often in studies, adverse childhood experiences are measured by adding them all together, i.e., an increasing total score increases the risk of developing various diseases, and at the same time increases the use of psychoactive substances [40]. However, ACEs have a very wide spectrum of human experiences, i.e., from emotional neglect or parental divorce to frequent experience of violence or even sexual violence. When assessing these impacts, it is increasingly suggested that these experiences are not equivalent and should be measured separately rather than as a cumulative total score. Previous research indicates that the use of a total ACE score may obscure important differences, as emerging studies have identified more specific associations between particular types and timing of adversity and patterns of psychoactive substance use [64]. Although research adopting this approach remains relatively limited, the present study sought to contribute to this area by examining how individual adverse childhood experiences are associated with the severity of psychoactive substance use in young adulthood [39,65]. The findings of the current study, together with the existing literature, suggest that there may be meaningful differences between direct and indirect forms of adversity. This consideration informed the decision to focus on direct experiences in the present analysis, while excluding indirect factors such as parental mental illness, substance use, divorce or bereavement, and imprisonment. According to our current study results, the possible, prognostic model of ACEs and alcohol use severity explains 13% of the variance, and only a few ACE components reach statistical significance, i.e., peer physical bullying, physical maltreatment, and sexual abuse. The results of physical maltreatment, also referred to in the literature as physical abuse, are in line with the data obtained by other researchers [58,66,67,68,69]. However, some studies did not find such a connection [70]. Like other researchers [54,69,71], we found a connection between childhood sexual abuse and alcohol use severity. Alcohol is a psychoactive substance belonging to the group of depressants, i.e., a large proportion of those with alcohol use disorder also suffer from depressive symptoms [72], and use alcohol as a means of self-medication until addiction develops over time. According to other authors’ results, victims of sexual abuse also show links to an increased risk of developing depression [73].

Although the current study did not examine physiological mechanisms, such as inflammatory markers or hypothalamic–pituitary–adrenal (HPA) axis functioning, the findings may be interpreted within a broader psychoneurobiological context. HPA axis may have a strong effect on substance use disorder (SUD) if we look at it through the lens of the use of psychoactive substances. This could be explained from an interdisciplinary point of view, that stress is not only caused by environmental factors such as adverse childhood experiences. But there is a broader concept involving biology-based mechanisms, such as oxidative stress, which can be caused by trauma, intoxication, unhealthy food, high blood sugar levels, polluted air, UV radiation, etc. [49,74]. Oxidative stress is associated with alterations in the HPA axis [75]. Thus, from an interdisciplinary point of view, it is possible that the increased use of psychoactive substances may not be caused only by adverse childhood experiences but, for example, by poor nutrition or neurodevelopment.

There is a possibility that the obtained findings that do not reflect the findings of other studies—i.e., lower alcohol consumption is predicted in those respondents who experienced more emotional abuse, and higher non-verbal emotional abuse predicts lower marijuana use—are due to suppression effects when various types of adverse experiences are used simultaneously in a single model. While we did not detect any collinearity in the models, we hypothesize that harsher adverse experiences (e.g., witnessing or experiencing violence) likely imply that there are also softer forms of adverse experiences (e.g., emotional abuse, non-verbal emotional abuse) present. Thus, when we account for all of the shared variance among the various forms of adverse experiences investigated in the present study, the variance that is unique to emotional abuse and non-verbal emotional abuse has a paradoxically reversed relationship with the dependent variable. This, in essence, does not show that emotional abuse could be a preventative measure for substance use, but, if our assumption is correct, rather implies that most forms of adverse experiences will also go together with emotional abuse, which in itself is an important finding. In practical terms, future research could perhaps focus more on the harsher adverse experiences while using verbal abuse and similar adverse experiences for supplementary analyses.

Several limitations of this study should be acknowledged. First, the use of convenience sampling could limit the representativeness of the sample and may introduce selection bias. In addition, the study group was characterized by a sex imbalance—many of whom were students—and this could restrict the generalizability of the findings to the broader population. Furthermore, we should pay attention to the fact that in the Lithuanian context, alcohol consumption is a cultural and completely normalized phenomenon, practiced by the vast majority of young people. At the same time, all other psychoactive substances except nicotine products are illegal, which is why respondents are likely to consume significantly less psychoactive substances. If they do consume psychoactive substances, they may be afraid to disclose it even in an anonymous survey. This might have affected the results of our prognostic model.

## 5. Conclusions

The findings of our study suggest that adverse childhood experiences may not be qualitatively equivalent and, therefore, may not be evaluated only as a cumulative risk score. Our results revealed that separate ACE evaluations, instead of aggregate calculation of ACEs, may be useful to understand better which specific negative experiences have the greatest impact on subsequent use of psychoactive substances. The regression models explain only a small portion of the variance, which suggests that other factors may contribute to a larger share. Additionally, the relatively small effect sizes observed in our analyses also suggest that ACEs may represent only one part of a broader set of factors contributing to substance use. Further research is needed to identify additional determinants and mechanisms.

## Figures and Tables

**Table 1:** Descriptive statistics of all variables used in analyses.

	Missing	M	SD	S	K	1	2	3	4	5	6	7	8	9	10	11	12
1. Alcohol abuse (AUDIT)	106	6.2279	5.744	1.985	5.227	—											
2. Cannabis abuse (CUDIT)	571	9.0828	8.280	1.199	0.506	0.252 **	—										
3. Hard drug abuse (ASSIST)	559	7.7962	9.789	1.449	1.307	0.417 ***	0.406 ***	—									
4. Emotional abuse	7	1.0730	1.092	0.960	0	0.149 ***	0.021	0.115	—								
5. Sexual abuse	7	0.0955	0.321	5.946	48.055	0.264 ***	0.224 **	0.343 ***	0.307 ***	—							
6. Non-verbal emotional abuse	7	1.0169	0.864	0.789	−0.234	0.166 ***	0.023	0.179 *	0.672 ***	0.349 ***	—						
7. Emotional neglect	7	1.0951	1.041	0.778	−0.294	0.153 ***	0.148	0.347 ***	0.568 ***	0.281 ***	0.630 ***	—					
8. Physical neglect	7	0.6071	0.736	1.395	1.256	0.166 ***	0.069	0.280 ***	0.404 ***	0.330 ***	0.387 ***	0.739 ***	—				
9. Physical maltreatment	7	0.5790	0.780	1.959	3.846	0.283 ***	0.127	0.194 *	0.678 ***	0.373 ***	0.562 ***	0.434 ***	0.377 ***	—			
10. Witnessing interpersonal violence	7	0.3337	0.680	2.595	6.811	0.197 ***	0.084	0.199 *	0.479 ***	0.363 ***	0.503 ***	0.337 ***	0.298 ***	0.473 ***	—		
11. Witnessing violence to siblings	7	0.1467	0.394	4.476	27.391	0.291 ***	0.213 *	0.313 ***	0.433 ***	0.578 ***	0.435 ***	0.305 ***	0.357 ***	0.608 ***	0.509 ***	—	
12. Peer emotional abuse	7	1.0324	1.093	0.920	−0.207	0.146 ***	0.086	0.178 *	0.516 ***	0.358 ***	0.509 ***	0.333 ***	0.214 ***	0.456 ***	0.377 ***	0.332 ***	—
13. Peer physical bullying	7	0.3746	0.658	2.562	6.986	0.248 ***	0.177 *	0.313 ***	0.409 ***	0.517 ***	0.412 ***	0.279 ***	0.280 ***	0.486 ***	0.366 ***	0.473 ***	0.700 ***

Notes. S—skewness, K—kurtosis, * *p* < 0.05, ** *p* < 0.01, *** *p* < 0.001.

**Table 2:** Regression analysis predicting hard drug use.

Predictor	Estimate	SE	t	*p*	Stand. Estimate
Intercept	5.2537	1.35	3.9045	<0.001	
Emotional abuse	−1.1483	1.01	−1.1380	0.257	−0.13613
Sexual abuse	2.5456	2.38	1.0714	0.286	0.12295
Non-verbal emotional abuse	−2.4844	1.44	−1.7237	0.087	−0.21856
Emotional neglect	4.3077	1.15	3.7478	<0.001	0.46921
Physical neglect	−0.0608	1.32	−0.0460	0.963	−0.00512
Physical maltreatment	−0.5913	1.22	−0.4835	0.629	−0.05688
Witnessing interpersonal violence	0.1092	1.19	0.0919	0.927	0.00902
Witnessing violence to siblings	2.7898	2.19	1.2738	0.205	0.16785
Peer emotional abuse	−0.7276	1.02	−0.7135	0.477	−0.08480
Peer physical bullying	2.8154	1.51	1.8649	0.064	0.23906

Notes. R^2^ = 0.252, F(10,146) = 4,92, *p* < 0.001. N = 157.

**Table 3:** Regression analysis predicting cannabis abuse.

Predictor	Estimate	SE	t	*p*	Stand. Estimate
Intercept	9.2453	1.286	7.1908	<0.001	
Emotional abuse	−1.2752	1.057	−1.2059	0.230	−0.15563
Sexual abuse	2.0667	2.083	0.9920	0.323	0.13186
Non-verbal emotional abuse	−3.1008	1.407	−2.2036	0.029	−0.31143
Emotional neglect	3.4491	1.129	3.0558	0.003	0.45825
Physical neglect	−2.3437	1.292	−1.8141	0.072	−0.23298
Physical maltreatment	0.2532	1.179	0.2149	0.830	0.02837
Witnessing interpersonal violence	−0.4968	1.140	−0.4358	0.664	−0.04915
Witnessing violence to siblings	2.9327	2.134	1.3744	0.172	0.21767
Peer emotional abuse	0.0134	0.925	0.0145	0.988	0.00195
Peer physical bullying	1.4359	1.444	0.9944	0.322	0.14536

Notes. R^2^ = 0.143, F(10,134) = 2.23, *p* < 0.019. N = 145.

**Table 4:** Regression analysis predicting alcohol abuse.

Predictor	Estimate	SE	t	*p*	Stand. Estimate
Intercept	5.2572	0.377	13.927	<0.001	
Emotional abuse	−0.7045	0.333	−2.116	0.035	−0.13527
Sexual abuse	1.9394	0.858	2.261	0.024	0.11258
Non-verbal emotional abuse	−0.1403	0.405	−0.347	0.729	−0.02154
Emotional neglect	0.4033	0.376	1.071	0.284	0.07521
Physical neglect	−0.0671	0.468	−0.143	0.886	−0.00861
Physical maltreatment	1.4955	0.432	3.462	<0.001	0.21066
Witnessing interpersonal violence	0.3202	0.406	0.789	0.430	0.03873
Witnessing violence to siblings	1.3002	0.801	1.622	0.105	0.09220
Peer emotional abuse	−0.3374	0.307	−1.100	0.272	−0.06525
Peer physical bullying	1.0396	0.508	2.046	0.041	0.12342

Notes. R^2^ = 0.131, F(10,599) = 9.04, *p* < 0.001. N = 610.

## Data Availability

The data supporting the findings of this study are available from the corresponding author upon reasonable request.

## Abbreviations

The following abbreviations are used in this manuscript:

ACEs	Adverse childhood experiences
HPA	Hypothalamic–pituitary–adrenal axis
SUD	Substance use disorders
UV	Ultraviolet rays
